# Clinical, economic and organizational impact of pharmacist interventions on injectable antineoplastic prescriptions: a prospective observational study

**DOI:** 10.1186/s12913-020-4963-7

**Published:** 2020-02-12

**Authors:** Céline Zecchini, Thi-Ha Vo, Sébastien Chanoine, Marion Lepelley, Mathieu Laramas, Aude Lemoigne, Benoît Allenet, Isabelle Federspiel, Pierrick Bedouch

**Affiliations:** 1 Centre Hospitalo-Universitaire Grenoble Alpes, Pôle Pharmacie, F-38000 Grenoble, France; 2 CNRS, TIMC-IMAG, UMR5525, F-38000 Grenoble, France; 3 Pham Ngoc Thạch University of Medicine, Hochiminh, V-70000 Vietnam; 4 University Grenoble Alpes, F-38000 Grenoble, France; 5 Centre Régional de Pharmacovigilance, F-38000 Grenoble, France; 6 Centre Hospitalo-Universitaire Grenoble Alpes, Pôle Cancer et maladies du sang, F-38000 Grenoble, France

**Keywords:** Pharmaceutical analysis, Chemotherapy, Onco-hematology, Pharmacist intervention

## Abstract

**Background:**

Pharmacists play a key role in ensuring the safe use of injectable antineoplastics, which are considered as high-alert medications. Pharmaceutical analysis of injectable antineoplastic prescriptions aims to detect and prevent drug related problems by proposing pharmacist interventions (PI). The impact of this activity for patients, healthcare facilities and other health professionals is not completely known. This study aimed at describing the clinical, economic, and organizational impacts of PIs performed by pharmacists in a chemotherapy preparation unit.

**Methods:**

A prospective 10-week study was conducted on PIs involving injectable antineoplastic prescriptions. Each PI was assessed by one of the four multidisciplinary expert committees using a multidimensional tool with three independent dimensions: clinical, economic and organizational. An ancillary quantitative evaluation of drug cost savings was conducted.

**Results:**

Overall, 185 patients were included (mean age: 63.5 ± 13.7 years; 54.1% were male) and 237 PIs concerning 10.1% prescriptions were recorded. Twenty one PIs (8.9%) had major clinical impact (ie: prevented hospitalization or permanent disability), 49 PIs (20.7%) had moderate clinical impact (ie: prevented harm that would have required further monitoring/treatment), 62 PIs (26.2%) had minor clinical impact, 95 PIs (40.0%) had no clinical impact, and 9 PIs (3.8%) had a negative clinical impact. For one PI (0.4%) the clinical impact was not determined due to insufficient information. Regarding organizational impact, 67.5% PIs had a positive impact on patient management from the healthcare providers’ perspective. A positive economic impact was observed for 105 PIs (44.3%), leading to a saving in direct drug costs of 15,096 €; 38 PIs (16.0%) had a negative economic impact, increasing the direct drug cost by 11,878 €. Overall cost saving was 3218€.

**Conclusions:**

PIs are associated with positive clinical, economic and organizational impacts. This study confirms the benefit of pharmacist analysis of injectable antineoplastic prescriptions for patient safety with an overall benefit to the healthcare system.

## Background

Antineoplastic drugs are among the therapeutic classes the most involved in drug related death due to their narrow therapeutic index, their high level of toxicity and the frailty of treated patients [1, 2]. Considered as high-risk medications, they require expertise from prescription to administration. Many actions are undertaken by pharmacists to ensure the safe management of injectable antineoplastics: centralized reconstitution, computerization, and strict prescription protocols. It is recognized that pharmacists play a crucial role in the safe use of antineoplastic drugs [3]. However, despite these security measures, there is always a residual risk of error, justifying the requirement for pharmaceutical analysis of injectable antineoplastic prescriptions.

In a hospital, the Chemotherapy Preparation Unit (CPU) aims at ensuring the safety of the preparation of injectable antineoplastic drugs under the supervision of a pharmacist. This centralized service can implement the systematic pharmaceutical analysis of injectable antineoplastic prescriptions to detect Drug Related Problems (DRPs), and to carry out Pharmacist Interventions (PIs) in collaboration with prescribers [3, 4]. A DRP is defined as an event or circumstance involving drug therapy that actually or potentially interferes with desired health outcomes [5]. A PI was defined as any action initiated by a pharmacist that directly resulted in a change in a patient’s therapeutic management [6, 7]. If accepted, a PI leads to the modification of patient’s therapeutic management [6].

Such modifications may have clinical consequences for patients: in the most severe cases PIs can prevent hospitalization for chemotherapy toxicity, permanent disability such as renal failure, or paresthesia, especially by detecting dosage problems or contraindications [8–10]. In others cases PIs can improve quality of life, patient satisfaction and adherence with counseling [11]. Taking into account the high direct cost of injectable antineoplastics PIs may have economic impact with cost savings: by dosage adjustments or by drug waste minimization with rounding of drug dosages and selection of the most convenient vial size [12]. PIs can also generate cost avoidance due to the prevention of adverse drug events: according to a study, the mean cost avoidance generated by a PI was 166€ [13, 14]. PIs may have also organizational impacts: pharmacists can engage actions to improve the quality of care process from the perspective of healthcare providers to optimize preparation and administration workflow [15, 16].

**Fig. 1:**
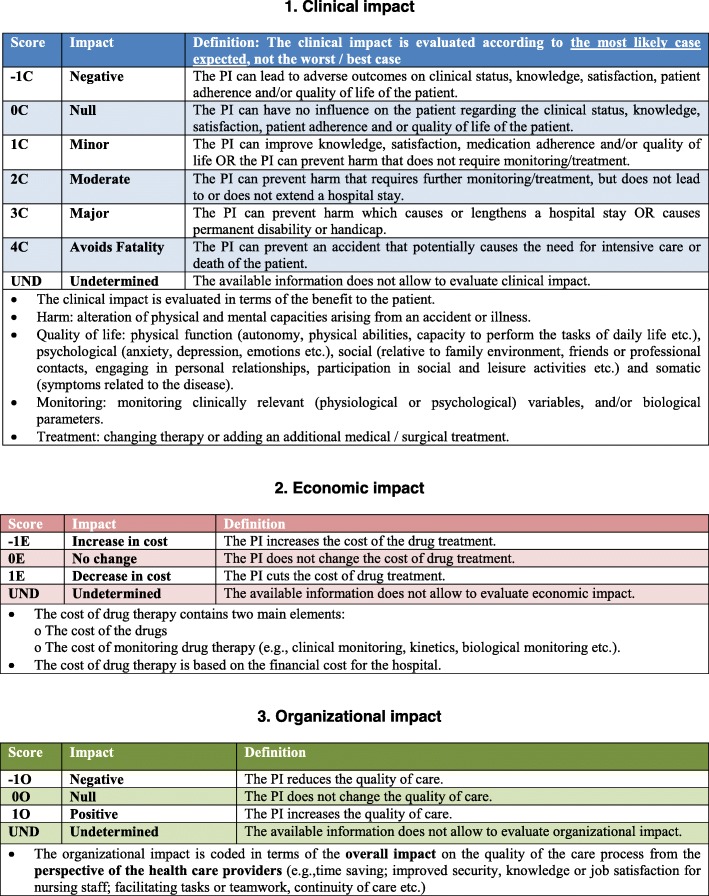
The CLEO tool

The French Society of Clinical Pharmacy (SFPC) developed and validated a multidimensional tool for assessing the whole impact of PIs, named CLEO (**CL**inical, **E**conomic, and **O**rganizational) (Fig. 1) [17, 18]. The CLEO tool includes three independent dimensions to evaluate clinical, economic and organizational impact of PIs to describe the whole impact of PI. The clinical dimension focuses on impact related to the patient’s well-being from the patient’s perspective: averted damages, improved quality of life, and improved adherence. The economic dimension assesses the immediate impact of the PI on the current costs of therapy from the institution’s perspective. The organizational dimension evaluates the impact on the process of care, focusing on the view of the health care professionals: reduced time expenditures, decreased work load, improved work place safety, and simplified collaborations. This tool was also translated and validated a the German version [19].

Other studies performed have evaluated clinical and economic impact of PIs, but to date, no oncohaematology studies have described the impact of PIs using a multidimensional approach, taking into account clinical, economic and organizational dimensions.

This study aims at describing the clinical, economic, and organizational impacts of PIs performed by pharmacists in the CPU of a French University Hospital.

## Methods

### Setting

A 10-week observational study in the CPU of the 2000-bed Grenoble Alpes University Hospital (France) was conducted. About 40,000 injectable antineoplastic preparations are produced per year.

A Computerized Provider Order Entry (CPOE) system is used for the prescription of injectable antineoplastics. Prescriptions are made by senior physicians or medicine residents according to standardized protocols that have been previously validated by both pharmacists and physicians. These protocols include: drugs, doses, administration modalities (route, infusion time, solvent), and required premedication. The protocol can concern the prescription of only one injectable antineoplastic or a combination of injectable antineoplastics, for one or more days. When the prescription has been electronically signed, it’s automatically transmitted in the software module for pharmacist analysis and preparation.

The prescription is analyzed at the CPU by a senior pharmacist or a trained pharmacy resident prior to administration. For each new prescription, pharmaceutical analysis includes: adequacy of the prescription with the treatment determined during a multidisciplinary cancer board, conformity of the prescribed protocol with the diagnosis and guidelines, doses, and appropriate patient’s characteristics. Pharmacists access the patient’s medical history through the hospital’s electronic medical record system. For prescription renewal, pharmacists verified: the patient’s pathophysiological data, cycle duration and number of courses, the previous prescription (drug, dose reduction, comment, and biological data if necessary). The CPOE automatically calculates the body surface area and doses.

When a DRP is identified, the pharmacist (senior or resident) of the CPU calls the medical staff to propose a PI. Finally, when the PI has been discussed with the medical staff, a preparation sheet is sent to pharmacy technicians to prepare chemotherapies.

### Data collection

All consecutive PIs concerning injectable chemotherapy prescriptions in inpatients units (medical oncology and hematology) and outpatient units (medical oncology including thoracic and gastro-intestinal cancer, hematology, radiotherapy) were collected, leading to 237 PIs relating to 185 patients over ten weeks.

PIs were retrospectively recorded using the electronic medical record system, generating a information report including: patients’ characteristics (sex, age, weight, height and body surface area), medical history, cancer drugs and cancer protocols used, a description of the DRP and PI according to the classification of the SFPC, and whether or not it was accepted by the physician [7]. These anonymized reports were provided to expert panels for assessment.

### Assessment of the impact of pharmaceutical interventions

Using the CLEO tool, the clinical, economic and organizational consequences of each PI was assessed by consensus in one of four multidisciplinary expert panels (medical oncology/radiotherapy, hematology, hepato-gastroenterology, and pneumology) [17]. Each panel consisted of four people: a specialist physician, a pharmacovigilance expert, a clinical pharmacist and a CPU pharmacist. The CLEO tool has three independent dimensions: clinical impact of the PI from the patient’s perspective, economic impact of the PI from the hospital’s perspective, and organizational impact of the PI from the healthcare provider’s perspective (Fig. 1). Each dimension of the CLEO tool has several numeric levels, with both negative, zero, positive and values, and an open level “non-determined”. The CLEO tool and 12 examples of assessments were provided to the expert panel before any PI evaluation. After the presentation of each PI by the meeting moderator, each expert independently scored clinical, economic and organizational impacts of the PI prior to discussion to reach an expert consensus.

### Economic analysis

Quantifying cost savings related to direct antineoplastic costs was determined by a complementary evaluation to the CLEO tool. Cost savings were determined by listing all PIs with a positive economic impact according to the expert panel assessment using the CLEO tool (e.g. trastuzumab 6 mg/kg instead of trastuzumab 8 mg/kg). Similarly, increased costs were calculated from all PIs having a negative economic impact according to the CLEO tool (e.g. trastuzumab 6 mg/kg instead of trastuzumab 2 mg/kg). This quantitative evaluation of savings or additional costs was conducted from the hospital’s perspective. It took into account the cost of injectable antineoplastics including value added tax but excluded the cost of solvents, diluents, and sterile medical devices used for preparation. Costs were calculated only for the treatment course concerned by the accepted PI, assuming that a PI has an impact only on one course. Cost savings were calculated based on the real costs of the PI accepted by the prescriber.

### Statistical analysis

Descriptive data are presented with frequency and percentage or as means with standard deviation (SD). All statistical analyses were performed using Microsoft Office Excel 2007.

## Results

Over the 10-week period, three pharmacists and two pharmacy residents of the CPU analyzed 1989 prescriptions of injectable antineoplastic involving 5284 preparations for 759 patients in inpatient units or outpatient units. Among them, 200 prescriptions (10.1%) relating to 185 patients had DRPs leading to 237 PIs. Patients concerned by DRPs were of mean age 63.5 ± 13.7 years, mainly male and had solid tumors (Table 1).

**Table 1:** Characteristics of patients with DRP (*n* = 185)

Patients characteristics	n (%)
Age, mean (SD)	**63.5 (13.7)**
Sex (male)	**100 (54.1)**
**Diagnosis**	
**Solid tumor**	**150 (81.1)**
Breast Cancer	26 (14.1)
Colorectal cancer	26 (14.1)
Non-small cell lung cancer	22 (11.9)
Head and neck cancer	21 (11.4)
Ovarian cancer	10 (5.4)
Cancer of the pancreas	8 (4.3)
Neuroendocrine cancer	8 (4.3)
Bladder cancer	5 (2.7)
Small cell lung cancer	5 (2.7)
Cervical cancer	4 (2.2)
Oesophageal cancer	4 (2.2)
Glioma	2 (1.1)
Sarcoma	2 (1.1)
Thymus carcinoma	2 (1.1)
Anal cancer	1 (0.5)
Astrocytoma	1 (0.5)
Biliary cancer	1 (0.5)
Gastric cancer	1 (0.5)
Prostate cancer	1 (0.5)
**Hematologic disease**	**35 (18.9)**
Non Hodgkin lymphoma	11 (6.0)
Multiple myeloma	8 (4.3)
Acute leukemia	7 (3.9)
Hodgkin lymphoma	3 (1.6)
Myelomonocytic leukemia	2 (1.1)
Chronic lymphocytic leukemia	1 (0.5)
Glanzmann thrombasthenia	1 (0.5)
Hairy cell leukemia	1 (0.5)
Idiopathic thrombocytopenic purpura	1 (0.5)

### Drug-related problems AND nature of the pharmacist interventions

DRPs mainly concerned dosages: 41.4% (*n* = 98) were a supra-therapeutic dosage and 15.2% (*n* = 36) a sub-therapeutic dosage (Table 2). The causes of supra-therapeutic dosages were mainly failure to make dose reductions due to toxicity and overestimations of patient’s weight. Sub-therapeutic dosages were often related to a failure to update of serum creatinine level in the dose calculation of Carboplatin and also errors in patient’s weight. In 12.2% of cases (*n* = 29), a parameter was missing for analyzing the prescription; in such cases monitoring or an update of monitoring (e.g. creatinine clearance) was requested to the prescriber. For 11.0% (*n* = 26), the DRP was related to non-conformity with the guidelines, e.g. the prescribed therapeutic protocol did not correspond to the correct protocol, the previously prescribed chemotherapy regimen or the regimen recommended for the patient at the last multidisciplinary cancer board. Untreated indications concerned 9.7% of PIs (*n* = 23); this problem being due to the lack of prescription for a patient or the absence of a drug in the prescribed protocol. The PIs were: dose adjustment (52.7%), drug switch request (15.2%), drug addition (9.7%), drug monitoring (9.3%), drug discontinuation (8.0%), and administration mode optimization (5.1%) (Table 2).

**Table 2:** Types of DRPs and PIs

Drug related problems	n (%)
Supratherapeutic dosage	98 (41.4)
Subtherapeutic dosage	36 (15.2)
Drug monitoring	29 (12.2)
Non-conformity to guidelines	26 (11.0)
Untreated indication	23 (9.7)
Inappropriate timing of administration	9 (3.8)
Drug without indication	7 (3.0)
Contra-indication	4 (1.7)
Improper administration	2 (0.8)
Adverse drug reaction	1 (0.4)
Non-conformity of the drug choice compared to the Formulary	1 (0.4)
Treatment not received	1 (0.4)
**Interventions**	
Dose adjustment	125 (52.7)
Drug switch	36 (15.2)
Addition of a new drug	23 (9.7)
Drug monitoring	22 (9.3)
Drug discontinuation	19 (8.0)
Administration mode optimization	12 (5.1)
**Total**	**237 (100)**

**Fig. 2:**
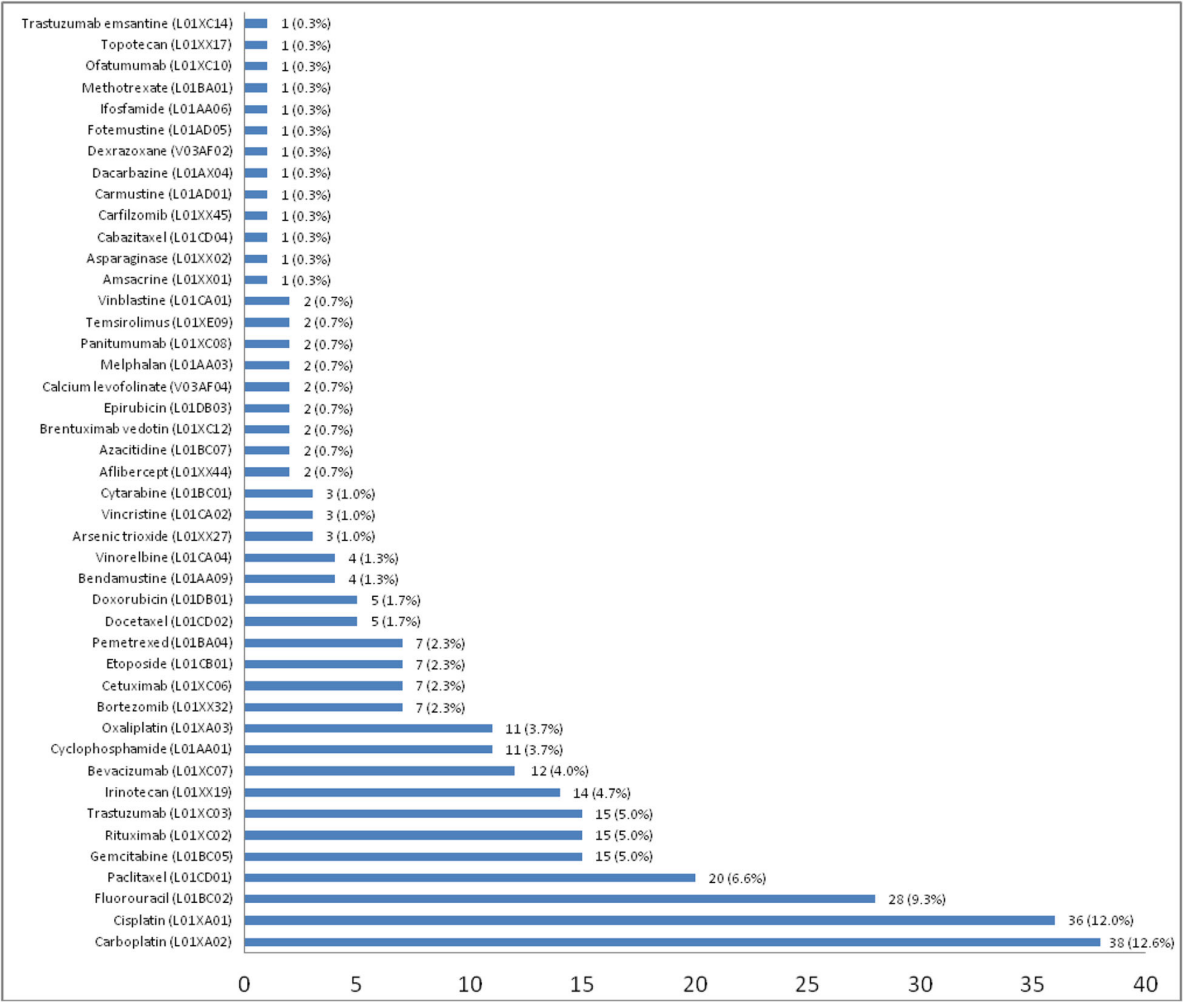
Anatomical Therapeutic Chemical (ATC) classification of chemotherapy drugs involved in Drug Related Problems

The main injectable antineoplastics involved in DRP were: Carboplatin (*n* = 38), Cisplatin (*n* = 36) and Fluorouracil (*n* = 28) (Fig. 2).

Most PIs (*n* = 179; 75.5%) were accepted by prescribers (refusal rate: 24.5%).

### CLINICAL, ECONOMIC AND ORGANIZATIONAL IMPACT OF PIs

The multidimensional impact was determined for 237 PIs using the CLEO tool.

**Fig. 3:**
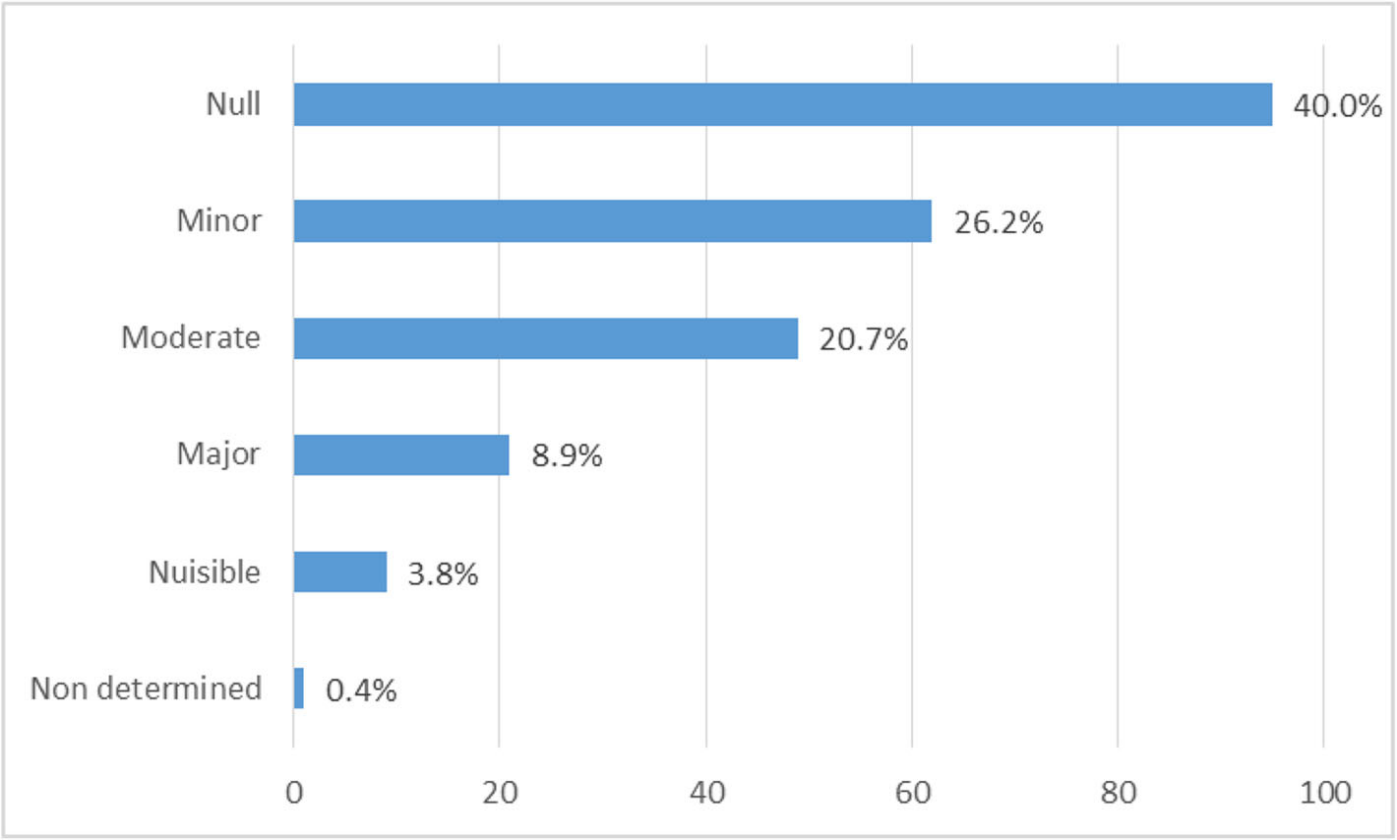
Clinical impact of Pharmacist’s Interventions

Overall, 21 PIs (8.9%) were considered to have had a major clinical impact, which could prevent hospitalization, the prolongation of hospitalization or permanent disability. Experts determined that neurological, hematological, renal, gastrointestinal and skin toxicities were avoided, but no PI was considered to have avoided a lethal accident. The list of these 21 PIs were summarized in Table 3. The majority of these PIs required dose adjustment. All PIs with major clinical impact were accepted by the prescribers. Experts considered that 49 PIs (20.7%) had a moderate clinical impact, which could prevent harm that would have required further monitoring or treatment, 62 PIs (26.2%) had a minor clinical impact that would have required further monitoring/treatment. While 95 PIs (40.0%) had no clinical impact, the panels considered 9 PIs out of 237 (3.8%) as potentially harmful; these PIs were all rejected by prescribers. For one PI (0.4%) the clinical impact was not determined because the committee had insufficient available information (Fig. 3).

**Table 3:** Description of PIs with a major clinical impact (*n* = 21)

Unit	DRP	Drug(s)	PI	Description	Economic impact	Organizational impact
Hepato-gastro enterology day care unit	Non conformity to guidelines	**Panitumumab**	**Drug switch**	Course number 1: Prescription VECTIBIX / FOLFOX instead of AVASTIN FOLFOX (RAS analysis in progress)	1	0
Hepato-gastro enterology day care unit	Non conformity to guidelines	**Panitumumab**	**Drug switch**	Course number 1: Prescription VECTIBIX / FOLFOX instead of AVASTIN FOLFOX (RAS analysis in progress)	1	0
Oncology day care unit	Non conformity to guidelines	**Gemcitabine**	**Drug discontinuation**	Prescription signed and green light given for gemcitabine but course should be canceled due to thrombocytopenia	1	1
Radiotherapy day care unit	Non conformity to guidelines	**Cisplatin**	**Dose adjustment**	Overdose: Prescription cisplatin 60 mg / m^2^ for 2 days, while the patient should not receive cisplatin on day 2	1	1
Pneumology day care unit	Contra indication	**Cisplatin**	**Drug discontinuation**	Prescription of an adjuvant cisplatin course for a patient having a clearance of creatinine 43 ml/min according to the CKD EPI formula	1	1
Oncology day care unit	Untreated indication	**Trastuzumab**	**Addition of a new drug**	Error in the protocol: missing one line in protocol on Taxotere Cyclophosphamide Trastuzumab	0	1
Oncology day care unit	Untreated indication	**Trastuzumab**	**Addition of a new drug**	Error in the protocol: missing one line in protocol on Taxotere Cyclophosphamide Trastuzumab	0	1
Hepato-gastro enterology day care unit	Supra-therapeutic dosage	**Fluorouracile** **Oxaliplatin**	**Dose adjustment**	Reductions of 50% of in 5 fluorouracil (5FU) and of 80% in oxaliplatin omitted in a patient with toxic ileitis to 5 FU in his medical records	1	0
Oncology day care unit	Supra-therapeutic dosage	**Cetuximab**	**Dose adjustment**	Expected reduction cetuximab 200 mg / m^2^ instead of 500 mg / m^2^ not appliedToxicity during previous treatments: folliculitis, xerosis	1	1
Oncology day care unit	Supra-therapeutic dosage	**Cyclophosphamide** **Doxorubicine** **Cisplatine**	**Dose adjustment**	Expected reductions of 80% for Cyclophosphamide and doxorubicin and of 66% for cisplatin omitted. During the intercure period: anemia, thrombocytopenia, non-febrile agranulocytosis, oedematous decompensation leading to emergency consultation	1	1
Oncology day care unit	Supra-therapeutic dosage	**Irinotecan**	**Dose adjustment**	Reduction of 80% for irinotecan omitted During the intercure period: Hospitalization for diarrhea during previous course	1	1
Oncology day care unit	Supra-therapeutic dosage	**Paclitaxel**	**Dose adjustment**	Expected reduction to 80% paclitaxel not appliedToxicity: paresthesia of hands and feet prior to paclitaxel	1	1
Oncology day care unit	Supra-therapeutic dosage	**Cabazitaxel**	**Dose adjustment**	Expected reduction to 80% for carbazitaxel Hospitalization during previous course for deterioration in general condition and nausea	1	1
Oncology day care unit	Supra-therapeutic dosage	**Paclitaxel**	**Dose adjustment**	Expected reduction of 80% for paclitaxel omitted Toxicity: Feet paresthesia prior to paclitaxel	1	1
Oncology day care unit	Supra-therapeutic dosage	**Paclitaxel**	**Dose adjustment**	Expected reduction of 80% paclitaxel not appliedToxicity: Hands and feet paresthesia prior to paclitaxel	1	1
Oncology day care unit	Supra-therapeutic dosage	**Paclitaxel**	**Dose adjustment**	Expected reduction of 80% for paclitaxel not applied Toxicity during previous treatments: Neuropathy	1	1
Oncology day care unit	Supra-therapeutic dosage	**Docetaxel**	**Dose adjustment**	Expected reduction of 66% for not applied Toxicity during intercure: Edema of the lower limbs	1	1
Oncology day care unit	Supra-therapeutic dosage	**Vinorelbine**	**Dose adjustment**	Prescription of weekly navelbine at dosage of 25 mg / m^2^Medical history: Neutropenia grade IV with a weekly navelbine protocol	1	1
Oncology day care unit	Supra-therapeutic dosage	**Vinorelbine**	**Dose adjustment**	Expected reduction of 80% for navelbine Toxicity during intercure: Hospitalization for general condition alteration and febrile peak	1	1
Oncology day care unit	Supra-therapeutic dosage	**Gemcitabine**	**Dose adjustment**	Expected reduction of 80% for gemcitabine not applied Toxicity during previous treatment: Febrile neutropenia	1	1
Oncology day care unit	Supra-therapeutic dosage	**Cisplatin** **Fluorouracile**	**Dose adjustment**	Course number 1: expected reductions because of asthenia and undernutrition by 80% for cisplatin and fluorouracile not applied	1	1

Regarding organizational impact, 165 PIs (67.5%) were evaluated as having a positive impact on the quality of patient management, and 71 (30.0%) and 6 PIs (2.5%) as having a null or unfavorable impact, respectively. Forty-one PIs were considered as having no clinical or economic impact but only a favorable organizational impact. These PIs most often concerned a date or hospitalization unit errors. These changes were requested in order to facilitate the work of healthcare providers in charge of the patient, allowing them to see the prescription at the right date for the correct care unit and authorizing them to validate electronically the administration of the chemotherapy.

Concerning economic aspects, according to the expert assessment using the CLEO tool, 105 PIs (44.3%) were assessed as having a positive impact (resulted in reduced cost of antineoplastic drugs), 94 PIs (39.7%) had null economic impact and 38 PIs (16.0%) a negative economic impact (increasing the cost of antineoplastic drugs). PIs accepted by prescribers with negative economic impact increased the direct drug costs by 11,878 € and PIs accepted by prescribers with positive economic impact represented a saving of 15,096 € (Table 4).

**Table 4:** Classification of Economic Impact and Quantifying of Cost Saving

Economic impact	Number of interventions (%)	Costs
−1: Negative	38 (16.0)	11, 878 €
0: Null	94 (39.7)	0 €
+ 1: Positive	105 (44.3)	15,096 €
**Total benefit**		**3218 €**

Through pharmaceutical analysis, the final cost saving was of 3218 € over the study period. Drug cost saving for a year would thus be 16,731 € for our hospital. For PIs accepted by prescribers and having a positive economic impact, the average saving per accepted PI was 181 ± 451 €, ranging from 0 € to 2019 € (median: 5.3 €; IQR: 15.9 €). These PIs were related to dose adjustments (*n* = 61), drug switch requests (*n* = 11) and drug discontinuations (*n* = 10). For PIs accepted by prescribers having a negative economic impact, the average additional cost per accepted PI was 439 ± 835 €, ranging from 0 € to 3262 € (median: 21.9 €; IQR: 507.2 €). These PIs were related to dose adjustments (*n* = 15), drug additions (n = 6), drug switch requests (*n* = 5) and administration mode optimization (n = 1).

**Fig. 4:**
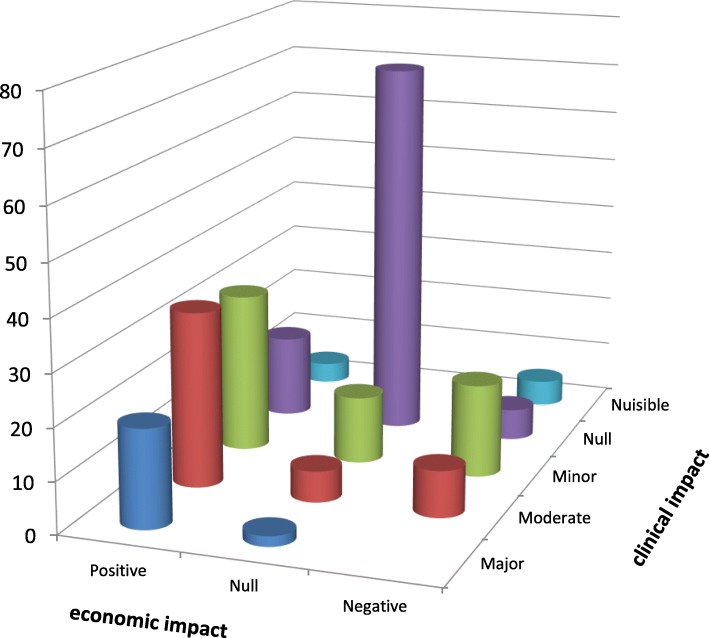
Combination of clinical and economic impact levels of PIs

PIs with a major, moderate and minor clinical impact predominantly had a positive economic impact with respectively 90.5, 69.4 and 50.0% positive economic impact (Fig. 4). No PI with major clinical impact had a negative economic impact. Seventy-three PIs had no clinical and economic impact, nevertheless 42 of them (57.5%) had a positive organizational impact (Fig. 4). Finally, few PIs (12.7%) had no impact on the 3 dimensions.

## Discussion

This study shows that DRPs are commonly encountered in injectable antineoplastic prescriptions. PIs related to DRPs significantly improve prescriptions in terms of clinical, economic and organizational dimensions: 55.8% of PIs had a positive clinical impact, 67.5% PIs had a positive impact on patient management from the healthcare providers’ perspective and a cost saving of 3218 € on drugs was realized over the study period.

### Findings

Previous studies have reported PIs as being needed for 1.5 to 27.6% of injectable antineoplastic prescriptions [20–24]. This wide range can be explained by organizational differences: in a center without computerized prescriptions, 27.6% of prescriptions required a PI [24]. A DRP rate of 3.0% was found in an establishment in which prescriptions were based on standardized pre-printed prescriptions and a listing of protocols [22]. Recent studies in settings with CPOE found a PI rate lower than 2% [20, 21]. In our study, the PI rate of 10.1% was quite high despite computerized prescriptions and use of protocols. A large proportion of prescriptions established by residents in medicine and the lack of update of creatinine values in the CPOE system may explain this finding.

Our results confirm those of previous studies which showed that overdosages and under-dosages are the most frequently encountered problems [20, 21, 24]. Consequently, in more than half of cases, PIs requested a dose adjustment. Monitoring problems is the third most commonly encountered DRP, mainly related to creatinine value updates required for platinum compounds. In agreement with previous studies, most PIs were formulated for two drugs: Carboplatin (*n* = 39) and Cisplatin (*n* = 36) [22]. This result can be explained by the risk of renal toxicity of platinum compounds requiring regular dose adjustment or leading to contra-indication.

In our study, PIs were accepted by senior physicians and medicine residents in 75.5% of the cases. This acceptance rate is close to that usually observed in cancerology and in others areas of clinical pharmacy [21, 25].

This study shows that the pharmaceutical analysis of injectable antineoplastic prescriptions has a positive clinical impact: 55.8% of PIs were considered to have a positive clinical impact. Our results are in accordance with the literature, even if the use of different tools does not allow us to directly compare our results with the previous studies. Knez et al. found that 48% of PIs involving injectable antineoplastic prescriptions were clinically very significant, another study in a large chemotherapy preparation unit reported that 50.4% of PIs were considered to be clinically significant [8, 23].

In our study, no PI was considered to have avoided a potentially lethal effect for the patient, while some other studies have reported PIs performed by pharmacists in a CPU that potentially avoided patient death [21, 23, 26]. In these studies, PIs with life-saving impact were essentially related to overdose prescription problems of 3-to 50-fold the theoretical doses or cases of co-prescription. During the period studied in our hospital there was no case of such large overdosage as that reported elsewhere, possibly due to safer procedures including computerized prescriptions and standardized protocols.

Our choice of using the CLEO tool enabled us to identify PIs with a negative clinical impact. Nine PIs were considered by the expert panels to have a negative clinical impact. For 5 of them, the inappropriateness of the PI was due to changes in patient’s clinical status that had not been saved into the electronic medical record system and therefore was unknown to pharmacists before their intervention.

Our study suggests that the pharmaceutical analysis of injectable antineoplastic prescriptions has a positive economic impact from hospital’s perspective by reducing direct drug costs: 44.3% of PIs decreased the cost of patient’s therapeutic management. These results are in accordance with previous studies [27–29]. In a study conducted in another French university hospital, focusing on the pharmaceutical analysis of injectable antineoplastic prescriptions, Nerich et al. found that 31.7% of PIs reduced direct drug costs [27]. In the same study, it was estimated that 1459 PIs carried out over a one-year period generated a saving of 25,136 € from hospital’s perspective. In our University Hospital, we estimated that savings would be 16,731 € over a year, somewhat less. However, it is difficult to compare results between these two studies, because the cost assessment is based on the purchase prices of injectable antineoplastic drugs, which differs between hospitals and over time. In our study, we assumed that a PI had an economic impact on a single course of chemotherapy, whereas other studies extrapolated the impact of a PI beyond a single course of the drug. Even taking the shortest chronological impact, pharmaceutical analysis of injectable antineoplastics is associated with a positive economic impact [30]. In another French study, the authors estimated that, over a year, chemotherapy-related drug errors could have resulted in an additional 216 days of hospitalization, and cost avoidance related to hospitalization and medication was estimated at 92,907€ if these errors had not been detected by pharmaceutical analysis [26].

A majority (67.5%) of PIs had a positive organizational impact. These PIs included changes in the date of prescription or in the hospital unit, and minimal dose adjustments. They were considered as having a positive organizational impact because the quality of the prescription was improved. For example in our organization, the CPOE automatically propose a date of prescription according to the date of the previous injection. If the next injection must be postponed by many days, the prescriber must manually modify the date proposed. If the date is not modified, the prescription will appear on the date originally planned. Despite the use of CPOE prescription errors remain persistent, this is a well-known problem, including for antineoplastics [31–33]. To our knowledge no other study took into account the organizational impact of PIs for healthcare providers. However, organizational impact was the most difficult of the three dimensions to assess, because of the many different indicators of care management (time savings, improved security, knowledge, job satisfaction, continuity of care etc.) and the need to take into account the different points of view of the various healthcare professionals (physicians, pharmacists, nurses).

### Strength and limitations

To the best of our knowledge, this is the first study describing the impact of PI for injectable antineoplastic prescriptions in a multidimensional way. Most tools focus on clinical outcomes and/or cost savings. However, some PIs that have no direct clinical or economic impact can benefit for the healthcare practitioners, for example, a PI that improves safety for nurses (e.g., the pharmacist suggests a change in the dosage form such that the nurse does not have to manipulate potentially toxic drugs). The organizational dimension of the CLEO tool aims to detect such effect. One could use all three dimensions, with a three-component code describing the entire impact of a PI.

As the CLEO tool does not quantify savings or additional costs, for the economic evaluation we used a complementary approach limited to the direct costs of drugs. The experts (specialist physicians and pharmacists) determined that, in some cases, neurological, hematological, renal, gastrointestinal and skin toxicities were avoided but the costs of treatments or additional days of hospitalization that would have been needed to manage these adverse drug events were not assessed.

## Conclusion

Pharmaceutical analysis of injectable antineoplastic prescriptions in a CPU is complementary of clinical pharmacy activities performed in oncology and hematology care units. Our study shows that pharmaceutical analysis of injectable antineoplastic prescriptions has clinical, economic and organizational impacts. The involvement of pharmacists reduces medication errors, some of which could have serious consequences for oncology patients due drugs toxicity. In a large hospital the centralization of the pharmaceutical analysis of injectable antineoplastic prescriptions presents additional economic and organizational interests, leading to PIs for dose adjustments, drug switches, or drug discontinuations that reduce the direct costs of drug management. The evaluation of this activity should be regularly conducted to assess the added value of pharmacists in improving quality of care for patients and for healthcare system and could also be used as indicator of pharmacist’s performance.

## Data Availability

The datasets used during the current study are available from the corresponding author on reasonable request.

## Abbreviations

ATC: Anatomical Therapeutic Chemical
CKD EPI: Chronic Kidney Disease EPIdemiology collaboration
CLEO: CLinical, Economic, and Organizational
CPOE: Computerized Provider Order Entry
CPU: Chemotherapy Preparation Unit
DRP: Drug Related Problem
IQR: Interquatile range
PI: Pharmacist Intervention
SD: Standard deviation
SFPC: French Society of Clinical Pharmacy
